# TriMag Microrobots: 3D‐Printed Microrobots for Magnetic Actuation, Imaging, and Hyperthermia

**DOI:** 10.1002/adma.202419708

**Published:** 2025-08-11

**Authors:** Liuxi Xing, Yulu Cai, Yapei Zhang, Kevin Mozel, Zhengxu Tang, Tengteng Tang, Vittorio Mottini, Saumya Nigam, Bryan R. Smith, Ian Y Lee, Tavarekere N. Nagaraja, Ping Wang, Xiangjia Li, Tong Gao, Jinxing Li

**Affiliations:** ^1^ Department of Biomedical Engineering and Institute for Quantitative Health Science and Engineering Michigan State University East Lansing MI 48824 USA; ^2^ Department of Aerospace and Mechanical Engineering Arizona State University Tempe AZ 85281 USA; ^3^ Department of Mechanical Engineering Michigan State University East Lansing MI 48824 USA; ^4^ Henry Ford Hospital Detroit MI 48202 USA; ^5^ Precision Health Program Michigan State University East Lansing MI 48824 USA; ^6^ Department of Radiology College of Human Medicine Michigan State University East Lansing MI 48824 USA; ^7^ Department of Chemical Engineering and Material Science Michigan State University East Lansing MI 48824 USA; ^8^ Department of Electrical and Computer Engineering Michigan State University East Lansing MI 48824 USA

**Keywords:** 3D printing, hydrogel, hyperthermia, magnetic particle imaging, microrobotics, nanoparticles

## Abstract

Microrobots hold immense potential in biomedical applications, including drug delivery, disease diagnostics, and minimally invasive surgeries. However, two key challenges hinder their clinical translation: achieving scalable and precision fabrication, and enabling non‐invasive imaging and tracking within deep biological tissues. Magnetic particle imaging (MPI), a cutting‐edge imaging modality, addresses these challenges by detecting the magnetization of nanoparticles and visualizing superparamagnetic nanoparticles (SPIONs) with sub‐millimeter resolution, free from interference by biological tissues. This capability makes MPI an ideal tool for tracking magnetic microrobots in deep tissue environments. In this study, “TriMag” microrobots are introduced: 3D‐printed microrobots with three integrated magnetic functionalities—magnetic actuation, magnetic particle imaging, and magnetic hyperthermia. The TriMag microrobots are fabricated using an innovative method that combines two‐photon lithography for 3D printing biocompatible hydrogel structures with in situ chemical reactions to embed the hydrogel scaffold with Fe_3_O_4_ nanoparticles for good MPI contrast and CoFe_2_O_4_ nanoparticles for efficient magnetothermal heating. This approach enables scalable, precise fabrication of helical magnetic hydrogel microrobots. The resulting TriMag microrobots, with the synergistic effects of Fe_3_O_4_ and CoFe_2_O_4_ nanoparticles, demonstrate efficient magnetic actuation for controlled movement, precise imaging via MPI for imaging and tracking in biological fluid and organs, including porcine eye and mouse stomach, and magnetothermal heating for tumor ablation in a mouse model. By combining these capabilities, the fabrication and imaging approach provides a robust platform for non‐invasive monitoring and manipulation of microrobots for transformative applications in medical treatment and biological research.

## Graphical abstract

TriMag microrobots are two‐photon polymerized hydrogel structures embedded with in situ synthesized Fe_3_O_4_ and CoFe_2_O_4_ nanoparticles, enabling magnetic actuation, high‐resolution magnetic particle imaging (MPI), and efficient magnetothermal heating for deep‐tissue biomedical interventions including tracking, navigation, and tumor ablation.

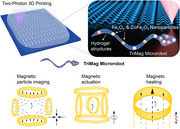

## 1 Introduction

Microrobots offer transformative potential in biomedical applications, including targeted drug delivery, detoxification, and minimally invasive surgeries. These miniaturized devices can access previously unreachable locations in the body, enabling cellular‐level procedures with remarkable precision and efficiency.^[^ ^1^, ^2^, ^3^, ^4^, ^5^, ^6^, ^7^ ^]^ Particularly, magnetic microrobots exhibit great promise for various in vitro and in vivo biomedical applications, such as locomotion in complex biological media,^[^ ^8^, ^9^, ^10^ ^]^ collective control and navigation,^[^ ^11^, ^12^, ^13^ ^]^ penetrating tumor or tissue for non‐invasive procedures,^[^ ^14^, ^15^, ^16^, ^17^, ^18^ ^]^ delivering a therapeutic drug to the target area.^[^ ^19^, ^20^, ^21^, ^22^, ^23^ ^]^ However, translating microrobotics from laboratory settings to safe clinical use remains a significant challenge.^[^ ^5^, ^6^, ^24^, ^25^, ^26^, ^27^, ^28^ ^]^ Key barriers include developing biocompatible materials for scalable and precise fabrication,^[^ ^29^, ^30^ ^]^ and integrating advanced imaging and actuation systems for visualization and navigation in deep biological tissue,^[^ ^3^ ^]^ as most of these devices are still visualized on a glass slide under optical microscopes.

A few imaging techniques have been explored for tracking microrobots in biological tissue, including magnetic resonance imaging (MRI), ultrasound, and optical coherence tomography (OCT).^[^ ^3^, ^21^, ^31^, ^32^, ^33^, ^34^ ^]^ Each of these methods has its advantages as well as limitations. MRI offers high penetration depth but is incompatible with magnetic actuation methods.^[^ ^35^, ^36^, ^37^, ^38^ ^]^ Ultrasound's relatively large wavelength restricts its resolution. At the same time, OCT's penetration depth is limited to around a few millimeters, making it challenging to image in very dense tissues, such as bone, where optical or acoustic waves are attenuated more.^[^ ^39^, ^40^, ^41^, ^42^, ^43^, ^44^ ^]^ Among imaging modalities, magnetic particle imaging (MPI) has emerged as a cutting‐edge technique due to its high sensitivity, real‐time imaging capabilities, and good spatial resolution potentially down to the micrometer level.^[^ ^45^, ^46^, ^47^, ^48^ ^]^ MPI operates by detecting the magnetization of superparamagnetic iron oxide nanoparticles (SPIONs) in response to external magnetic fields, creating high‐contrast images without interference from biological tissues. Unlike conventional imaging methods such as MRI or ultrasound, MPI directly visualizes magnetic tracers without generating any background noise, allowing deep tissue imaging with sub‐millimeter resolution.^[^ ^48^, ^49^, ^50^, ^51^ ^]^ These advantages position MPI as a promising tool for visualizing microrobots within deep biological tissue. Traditional MPI has focused on nanoparticle characteristics such as size, shape, and magnetization saturation to enhance signal sensitivity and imaging resolution.^[^ ^49^, ^52^, ^53^, ^54^ ^]^ Modifications, such as designing tailored SPIONs or incorporating Fe‐Co nanoparticles with graphitic carbon shells, have demonstrated improved imaging contrast.^[^ ^45^, ^47^ ^]^ However, these innovations often focused on passive nanoparticles mainly used for imaging, which lack maneuverability or functionality for targeted therapy.

In this study, we present a novel fabrication approach for hydrogel‐based magnetic microrobots by integrating two‐photon lithography 3D printing with in situ chemical reactions to produce magnetic nanoparticles. The resulting TriMag microrobots are composed of biocompatible poly(ethylene glycol) diacrylate (PEG‐DA) and pentaerythritol triacrylate (PETA) hydrogels embedded with Fe_3_O_4_ and CoFe_2_O_4_ nanoparticles. These TriMag microrobots synergistically integrate two types of magnetic nanoparticles to achieve triple functionalities: magnetic actuation, magnetic particle imaging, and rapid magnetothermal heating. We demonstrate that microrobots can be actuated by an electromagnetic field and imaged within dense biological tissues and animal bodies for both tracking and heating. With the potential clinical applications of MPI, the results highlight the significant potential of the MPI‐microrobot system, providing a robust platform for tracking, imaging, and real‐time monitoring of therapeutic interventions.

## 2 Results

### 2.1 TriMag Microrobots Nanofabrication

**Figure 1:**
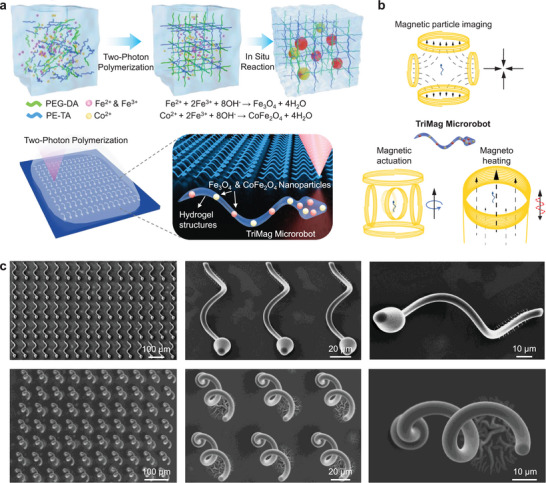
Fabrication and functionalization of TriMag microrobots. a) The schematic of the TPP fabrication process for the microrobot using a custom‐made photosensitive hydrogel, including the schematic of the 3D‐printed hydrogel and the chemical reaction to produce magnetic nanoparticles within the hydrogel. b) The schematic of the three magnetic operations of the TriMag microrobots, including magnetic imaging using an MPI scanner, magnetic actuation, and magnetic hyperthermia. c) Scanning electron microscope (SEM) images showing the top view and tilted view of an individual helical microrobot and a microrobot array used in this research. The displayed microrobot structures are fabricated with pure PEG‐DA/PE‐TA hydrogel, without functionalization, to demonstrate the hydrogel's printability using TPP fabrication.

The fabrication of microrobots is based on two‐photon polymerization (TPP) of a functional photosensitive hydrogel (**Figure** **1**), offering several advancements over current methods. Traditional TPP‐based microrobots, which incorporate nanoparticles, primarily rely on direct printing technology. In such a process, nanoparticles are mixed with 3D printing resins and polymerized under a laser beam for polymerization.^[^ ^55^, ^56^ ^]^ However, since most magnetic nanoparticles are either absorbing or scattering light, they can significantly reduce the transparency of the resin, which negatively impacts the efficiency of the two‐photon polymerization.^[^ ^55^, ^57^, ^58^ ^]^ Additionally, it is also challenging for the nanoparticles to form a stable and uniform suspension in the resin, leading to issues such as aggregation, which affect the printing outcome. Alternatively, post‐coating the 3D‐printed structures with magnetic thin films usually involves a costly cleanroom deposition process and also limits the amount of magnetic materials to only a thin layer^[^ ^28^ ^]^, which will affect the subsequent magnetic operation, imaging, or intervention. Our innovative approach addresses these challenges by utilizing an aqueous solution of iron (Fe) and cobalt (Co) ions, combined with a photosensitive hydrogel, for nanoscale 3D printing. The dissolved ions, rather than the nanoparticle dispersions, do not affect the solution's transparency, thereby maintaining the effectiveness of the laser beam during the two‐photon polymerization process. With the presence of metal ions, the 3D‐printed hydrogel structures can be functionalized through in situ chemical reactions. By adjusting the concentration of these ions in the precursor, we can control the weight or ratio of the generated nanoparticles.

Figure 1a illustrates the TriMag microrobot fabrication process, where the curing of the photosensitive hydrogel occurs at the focal point of the laser beam, resulting in high‐resolution structures. The hydrogel is based on the photopolymerization of biocompatible poly(ethylene glycol) diacrylate (PEG‐DA) and pentaerythritol triacrylate (PETA), here referred to as PEG‐DA/PE‐TA, with phenylbis(2,4,6‐trimethylbenzoyl) phosphineoxide (BAPO) as the photoinitiator. As a derivative of PEG, the PEG‐DA is widely used as a bio‐degradable medical material since the PEG component is susceptible to oxidation and degradation in biological environments,^[^ ^59^, ^60^ ^]^ while adding PETA prepolymer will increase the degree of crosslinking for improving the mechanical strength of the microrobot.^[^ ^57^, ^61^, ^62^, ^63^ ^]^ In biological fluids, hydrolysis of end group acrylate esters and oxidation of the ether backbone result in chain breaks of the crosslinked polymers.^[^ ^57^, ^64^, ^65^ ^]^ The degrading profile of the prepared PEG‐DA/PE‐TA hydrogel in various liquid environments (PBS, bovine serum, cerebrospinal fluid, and artificial gastric fluid) is shown in Supplementary Figure S1 (Supporting Information). After two‐photon printing, the hydrogel structures are exposed to hydroxide ions using NH_4_Cl or NaOH, and these Fe and Co ions react with the hydroxide ions to form Fe_3_O_4_ and CoFe_2_O_4_ nanoparticles,^[^ ^66^, ^67^, ^68^ ^]^ which are either adhered to the hydrogel surface or trapped inside the hydrogel network. Fe_3_O_4_ shows superparamagnetic properties and high magnetic susceptibility near the zero point during MPI imaging, making it an optimal MPI tracer for higher contrast and large magnetic force/torque when actuated by an external field.^[^ ^46^, ^47^ ^]^ On the other hand, due to its slow magnetic moment relaxation when compared with Fe_3_O_4_ nanoparticles, CoFe_2_O_4_ nanoparticles exhibited much higher efficiency for magnetothermal heating.^[^ ^68^, ^69^, ^70^ ^]^ Figure 1b demonstrates the triple magnetic operation of the resulting TriMag microrobots: actuation, imaging, and heating. The Fe_3_O_4_ particles provide strong performance in magnetic imaging, while the CoFe_2_O_4_ particles offer excellent magnetothermal effects when exposed to a high‐frequency oscillating magnetic field. Both types of nanoparticles also serve as effective magnetic actuation agents, allowing for precise control of the microrobots via an external magnetic field. Figure 1c presents scanning electron microscope (SEM) images of the 3D‐printed sperm‐mimicking PEG‐DA/PE‐TA hydrogel structures, shown both as an array and individually. These images highlight the scalable and precise printing capabilities of the hydrogel, as well as the high accuracy and relatively smooth surface achieved in the two‐photon printing process.

### 2.2 Morphological and Element Analysis of the TriMag Microrobots

**Figure 2:**
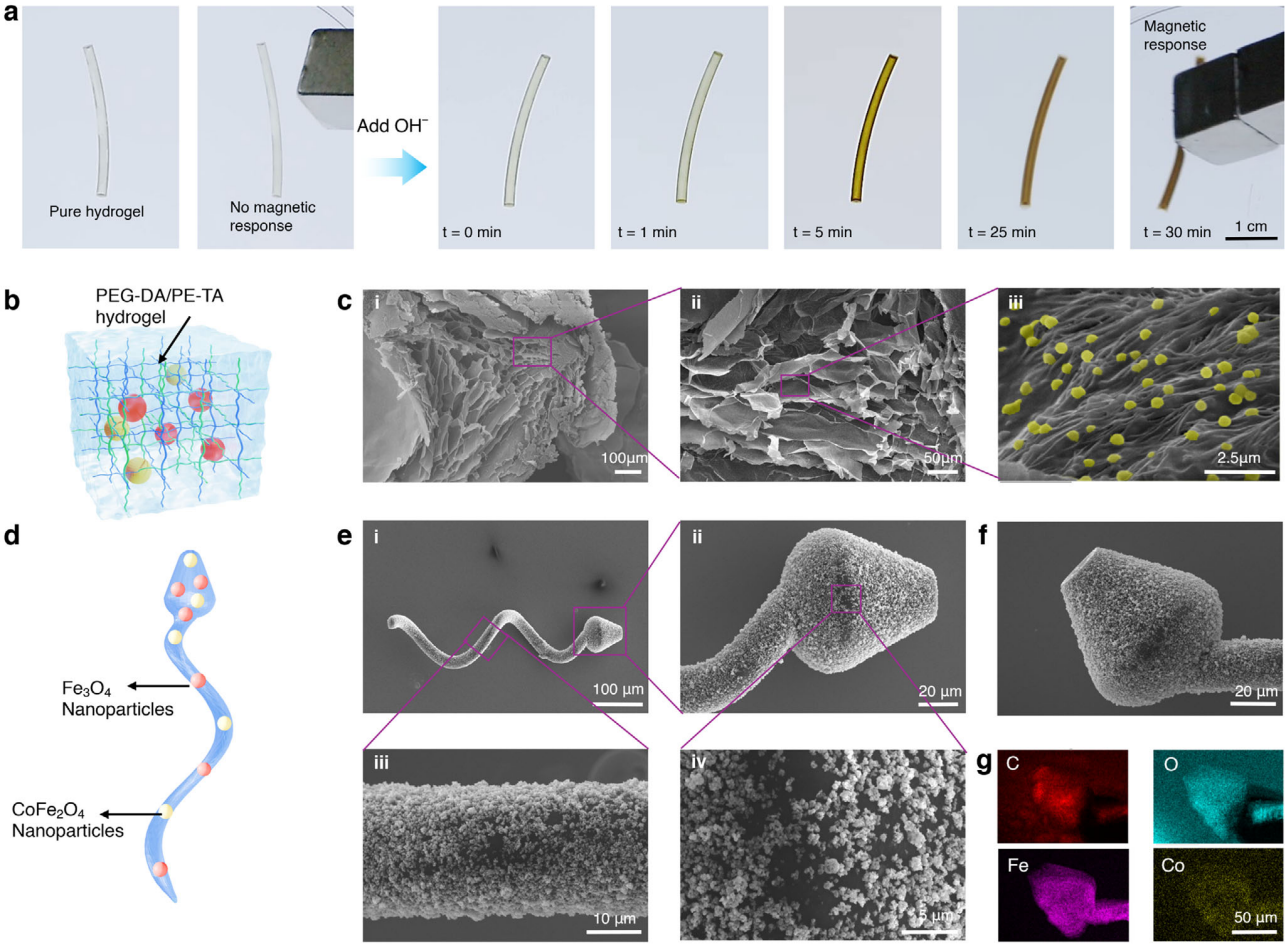
Morphological and elemental analysis of functionalized 3D‐printed TriMag microrobots. a) Visualization of the in situ reaction forming magnetic nanoparticles within the hydrogel matrix. Scale bar = 10 mm. b) A schematic illustration of the nanoparticles embedded within the hydrogel network. c) SEM images showing the generated nanoparticles accumulated inside a bulk‐crosslinked hydrogel structure. d) A schematic illustration of the 3D‐printed microrobot with nanoparticles distributed on its surface. e) Surface morphology of the microrobot with nanoparticles, visualized using SEM and EDX. The EDX analysis indicates the distribution of carbon, oxygen, iron, and cobalt on the microrobot.

To visualize the in situ reaction that forms magnetic nanoparticles on the hydrogel matrix, we first performed the chemical reaction on a bulk cross‐linked PEG‐DA/PE‐TA hydrogel fiber (**Figure** **2**a; Video S1, Supporting Information). The fiber changed color from transparent to iron‐like following the addition of a 10% NaOH solution (details in Experimental Section). The resulting hydrogel was then fixed using critical point drying for microscopic characterization. SEM images in Figure 2b,c show that the PEG‐DA/PE‐TA hydrogel forms a highly porous network, with nanoparticles decorating the polymer surface. SEM and Energy Dispersive X‐ray (EDX) mapping of the pure hydrogel, nanoparticles, as well as the surface and internal structures of the nanoparticle‐decorated hydrogel after reaction, are presented in Figure S2 (Supporting Information). The results indicate that the Fe_3_O_4_ and CoFe_2_O_4_ nanoparticles are readily synthesized on the hydrogel surface. Figure 2d shows the schematic of a TriMag microrobot, while Figures 2e,f demonstrate the surface morphology of the microrobot, where nanoparticles were successfully generated and grown on the polymer surface, forming the functional component of the entire structure. EDX analysis in Figure 2g shows the uniform distribution of iron and cobalt on the microrobot.

### 2.3 Microrobot System Design and Actuation

The helical magnetic microrobot is actuated by a rotating magnetic field generated through a 3‐axis Helmholtz coil system (Figure S3, Supporting Information). Inspired by the locomotion of flagellated bacteria such as *E. coli*, the corkscrew‐shaped microrobot has become a foundational design in micro/nanoscale propulsion since its initial proposal over a decade ago.^[^ ^71^, ^72^ ^]^ Following magnetic functionalization with superparamagnetic nanoparticles, the microrobot aligns with the external magnetic field, generating torque through the interaction between the field and the microrobot's average magnetization.^[^ ^5^, ^71^, ^73^ ^]^ As the orientation of the rotating field changes, a continuous magnetic torque is applied, causing the helical structure to spin. This rotational motion is transformed into forward propulsion through fluid, mimicking the motion of bacterial flagella. Depending on the helix's geometry, the microrobot can exhibit rolling (somersault‐like) or propulsive motion. The key control parameter in this system is the frequency of the rotating magnetic field, which determines the microrobot's synchronous rotation. In this steady state, magnetic torque balances the viscous drag torque, enabling stable and efficient locomotion. The helix angle and the length‐to‐diameter ratio of the microrobot primarily influence the propulsion efficiency. By adjusting the helix radius and pitch, the helix angle was optimized to ≈45°, a configuration that has been shown to minimize misalignment and maximize translational efficiency.^[^ ^28^, ^71^ ^]^ Furthermore, the microrobot's motion mode, speed, and direction can be precisely controlled by adjusting the frequency, amplitude, and orientation of the rotating magnetic field.^[^ ^13^ ^]^ Depending on the application, actuation can be achieved using either electromagnetic coils or permanent magnets. In our study, the Helmholtz system enabled flexible control of the microrobot in an open environment (Figure S3, Supporting Information).

**Figure 3:**
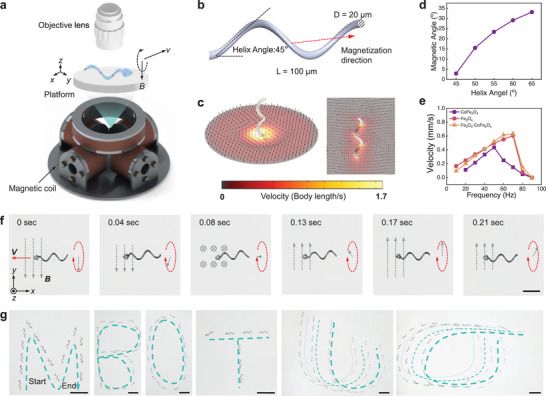
Actuation experiment of TriMag microrobot using 3‐axis magnetic actuation system. a) Schematic of the complete magnetic actuation system, including three pairs of electromagnetic coils, a container providing the liquid environment, and an objective lens representing the imaging system. b) Numerical model of the microrobot. c) CFD results showing the microrobot propulsion process in a liquid environment at a frequency of 50 Hz. d) The relationship between the misalignment angle (angle between magnetization direction and axis) and the helix angle. e) Frequency–velocity results for microrobots fabricated with different magnetic materials. f) The snapshot picture shows the details of the magnetic actuation. The motion energy comes from the rotating magnetic field, and the spiral motion of the tail provides the propulsion force. Scale bar = 50 µm. g) Trajectory control results for an individual microrobot and a microrobot swarm. Scale bar = 200 µm.

**Figure** **3**a shows the schematic of the complete microrobot actuation system, comprising three pairs of electromagnetic coils, a liquid container, and the imaging system (simplified to show only the objective lens). When a rotating magnetic field is applied, the microrobot spins and generates propulsion force along its axial axis, driving forward motion. Figure 3b,c presents the numerical model and simulation results of the microrobot (length 100 µm, suitable for most in vivo applications) at an actuation frequency of 50 Hz. At this frequency, the microrobot exhibits typical laminar dynamic properties in the low‐Reynolds‐number regime, with results applicable across scales from sub‐micrometer to millimeters.^[^ ^71^, ^74^, ^75^ ^]^

Figure 3d illustrates the relationship between misalignment angle and helix angle. As the helix angle increases, the magnetization direction deviates from the robot axis, complicating actuation and control. The helix angle was optimized to 45 degrees, which exhibits the lowest misalignment angle.^[^ ^28^ ^]^ The length‐to‐diameter ratio was also optimized for maximum propulsion efficiency.

To study the effect of magnetic nanoparticles on the propulsion performance of the TriMag microrobots, we investigated the velocity‐frequency dependence of microrobots functionalized with different magnetic nanoparticles (Figure 3e): Fe_3_O_4_ nanoparticles (with a prepared hydrogel precursor containing 5 × 10^−4^ M Fe^2+^ and 2 × 10^−3^ M Fe^3+^), CoFe_2_O_4_ nanoparticles (prepared with a hydrogel precursor containing 2 × 10^−3^ M Fe^3+^ and 5 × 10^−4^ M Co^2+^), and a combination of Fe_3_O_4_ and CoFe_2_O_4_ nanoparticles (prepared with a hydrogel precursor containing 5 × 10^−4^ M Fe^2+^, 2 × 10^−3^ M Fe^3+^, and 5 × 10^−4^ M Co^2+^) (detailed process in Experimental Section). Microrobots with the combined components (Fe_3_O_4_ and CoFe_2_O_4_ nanoparticles) and Fe_3_O_4_ nanoparticles only showed slightly faster speed. While the same frequency should generate the same speed for the helical microrobot, this speed difference may be due to the small magnetic field gradient within the magnetic coils. Due to their low saturation magnetization, microrobots with only CoFe_2_O_4_ nanoparticles showed the lowest velocity and reached the step‐out frequency beyond 50 Hz. Time‐lapse images in Figure 3f show that the rotational motion is transformed into forward propulsion through fluid, mimicking the motion of bacterial flagella. Figure 3g illustrates the trajectory control results of the TriMag microrobot with Fe_3_O_4_ and CoFe_2_O_4_ nanoparticles, showing an individual microrobot writing “MBOT” and swarm control with three microrobots operating simultaneously in open space, highlighting their precise motion control capabilities (Video S2, Supporting Information).

In this study, we investigated two of the commonly used magnetic nanoparticle materials. In future work, we will further explore the potential of emerging nanomaterials with enhanced MPI or magnetic hyperthermia performance. For example, substituting Co^2^⁺ with Mn^2^⁺ or Zn^2^⁺ may improve MPI sensitivity, incorporating minor FeCo alloys could enhance heating efficiency, etc.^[^ ^45^, ^76^ ^]^ Complex structures such as core–shell architectures or multicore clusters are also capable of optimizing magnetic behavior.^[^ ^77^, ^78^ ^]^ Additionally, the fabrication process can be refined to control particle size and morphology precisely. For instance, maintaining magnetic core sizes in the tens‐of‐nanometers range to achieve high magnetic moments while minimizing aggregation or blocking risks.^[^ ^45^, ^79^ ^]^ Morphology factors, such as multicore clusters, can enhance magnetization through interparticle interactions, while hollow structures may offer improved heating due to increased surface area.^[^ ^80^, ^81^, ^82^ ^]^ These advancements will pave the way for developing next‐generation nanoplatforms tailored for precise, efficient, and clinically translatable theragnostic applications.

### 2.4 MPI Characterization of Microrobot

**Figure 4:**
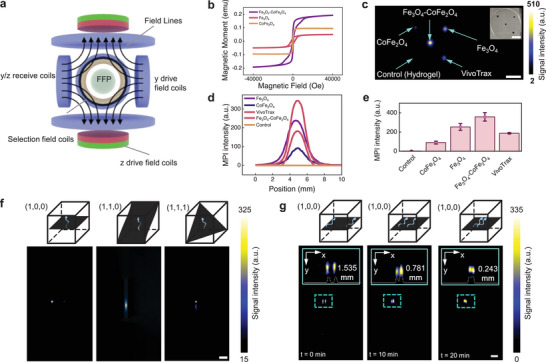
MPI characterization of material and microrobot. a) Fundamental diagram of the MPI system. b) Magnetization hysteresis loops of individual magnetic particle components and combined materials. c) MPI imaging of hydrogels containing different magnetic nanoparticles. Scale bar = 10 mm. d) MPI signal intensity showing the quantitative results for different materials. e) Statistical analysis of magnetic sensitivity for various materials, with sample size = 3, presented as mean ± SD. f) MPI imaging of an individual microrobot from different perspectives, highlighting the structure of a single microrobot. g) MPI imaging of two microrobots moving in close proximity to each other. f,g) Scale bar = 500 µm.

We then evaluated the imaging performance of the TriMag microrobots using MPI. Developing new nanoparticles for improved MPI imaging and magnetothermal heating is still an important research topic, as shown in recent works.^[^ ^45^, ^80^, ^83^ ^]^ While conventional Fe_3_O_4_ SPIONs are widely used for MPI, our microrobots incorporate two types of magnetic nanoparticles (Fe_3_O_4_ and CoFe_2_O_4_), which differ significantly in magnetic properties, aggregation behavior, and relaxation dynamics. It is therefore necessary to experimentally validate that MPI remains effective for imaging these heterogeneous materials with a sufficient signal‐to‐noise ratio and spatial resolution. As depicted in **Figure** **4**a, a basic diagram of the MPI system is shown, where the microrobot's magnetization changes as the field‐free line moves through its location, inducing a voltage in the receiver coil. We first measured the magnetization hysteresis loops of different magnetic hydrogels used in microrobot fabrication at room temperature (Figure 4b), including PEG‐DA/PE‐TA hydrogel, PEG‐DA/PE‐TA with Fe_3_O_4_ nanoparticles, PEG‐DA/PE‐TA with CoFe_2_O_4_ nanoparticles, and a hydrogel with both Fe_3_O_4_ and CoFe_2_O_4_ nanoparticles (same process as the above results). These results highlight the unique magnetic properties of the hydrogel with both Fe_3_O_4_ and CoFe_2_O_4_ nanoparticles, which exhibit superparamagnetic properties and high magnetic susceptibility near the zero point, making it an optimal MPI tracer for higher contrast and large magnetic force/torque when actuated by an external field.^[^ ^46^, ^84^ ^]^ The small circle on top of the loop proves the larger bulk anisotropy constant, indicating that its magnetothermal properties outperform common superparamagnetic iron‐oxide particles, benefiting from the Co component in the hydrogel.^[^ ^85^, ^86^, ^87^ ^]^

To evaluate their MPI contrast, the above magnetic hydrogel materials are fabricated into spherical shapes with a diameter of 1 mm and fixed in a 35 mm container using glue (Figure 4c). These are then imaged by the MPI scanner with a standard scanning profile. This quantitative result is confirmed in Figure 4d, where each sample specimen was independently tested three times to minimize interference, and the analytical results are shown in Figure 4e. The MPI image reveals the strongest intensity for Fe_3_O_4_, Fe_3_O_4_‐CoFe_2_O_4_ combination, and the commercial Vivotrax materials based on Fe_3_O_4_, while the CoFe_2_O_4_ and control groups (pure gel without functionalization) are much weaker on the MPI scanner.

To localize the position of the helical microrobot, the microrobot was used in the open area actuation experiment (500 µm) and fixed at the center of a D35 mm × 10 mm Petri dish with 0.6% agarose. A full 3D MPI scanning sequence is applied, and the imaging of the microrobot from a specific perspective is obtained, shown in Figure 4f. The strongest signal appears on the (110) face, which corresponds to the cylindrical longitudinal section of the microrobot. This indicates that the spatial position of the microrobot can be observed. The microrobot appears as a blurred dot on the (100) face, while on the (111) face, the microrobot is seen as a magnetized head with a helical tail. However, the signal intensity is weaker than that observed on the (110) face. These images demonstrate the MPI's potential to determine the microrobot's orientation.

To further assess the spatial resolution, the first microrobot is sealed in the Petri dish and positioned at the center of the MPI scanner's field of view. The second microrobot, made with the same formula, is mounted in a hydrogel and moved closer to the centered sample in the radial direction. Images of the two microrobots are taken using the same 2D sequence. The distance between the two microrobots is gradually decreased until the reconstruction can no longer distinguish the two samples. The distance between the robots is measured using an integrated camera during each scan. As shown in Figure 4g, the MPI imaging demonstrates that the two microrobots approach each other from 1.535 to 0.243 mm, after which the images of the two robots begin to merge. During this experiment, as the distance between the two robots decreases, they attract each other and move inside the hydrogel due to magnetic interaction. This finding suggests that MPI can provide orientation‐sensitive information, enabling identification of the microrobot's pose in space. Such insight is valuable not only for optimizing the design of microrobots for visibility under MPI, but also for establishing real‐time feedback mechanisms for orientation tracking in future closed‐loop control systems. The ability to distinguish two microrobots is also important to control and navigate multiple microrobots or microrobot swarms.

### 2.5 MPI Imaging of Microrobot in Biological Tissue

**Figure 5:**
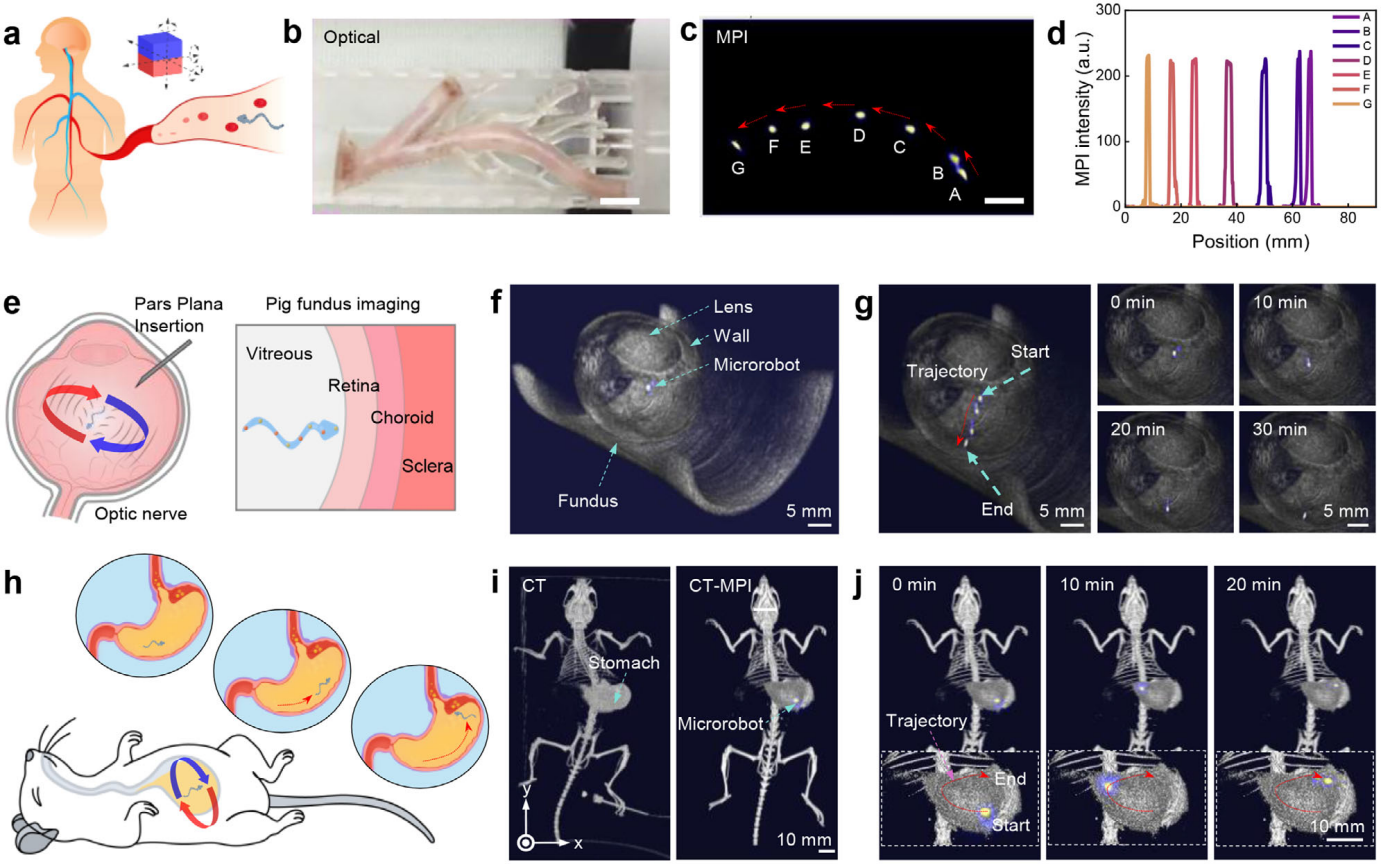
MPI imaging of TriMag microrobot in biological tissue. a) Schematic of the microrobot moving within a constrained in vivo environment. b) Experimental setup for the microrobot in a blood vasculature phantom filled with porcine brain tissue. c) Sequential images showing the motion of the microrobot at seven distinct positions within the phantom. b,c) Scale bar = 10 mm. d) Intensity measurements at various points in the image; the peak points of the data represent the microrobot's motion center. e) Schematic illustration of microrobot actuation within a porcine eye for posterior segment access. The microrobot is injected into the eyeball with a pipette, then actuated toward the fundus. h) The CT‐MPI tracked microrobot moves in the porcine eyeball; time‐lapse CT‐MPI trajectory of the microrobot is shown inside the eyeball, positions are tracked every 10 min. e) Schematic of an ex vivo experiment demonstrating the microrobot's motion in a mouse stomach under MPI guidance. f) The CT‐MPI tracked the microrobot's moves in a mouse stomach. The stomach and microrobot are observed by CT and MPI imaging, respectively. After being injected into the deep position of the stomach, the microrobot is moved inside by a magnetic field, following a desired “C” shape trajectory.

**Figure** **5**a illustrates a schematic of a microrobot navigating an in vivo constrained environment. To demonstrate the possibility of deep tissue imaging, a blood vasculature‐mimicking phantom, which was filled with fresh porcine brain tissue, was used for magnetic particle imaging (MPI) evaluation. To move in the tissue, the 3D‐printed microrobots were actuated by combining an oscillating magnetic field with a strong magnetic gradient. During the actuation process, the microrobot was imaged at seven distinct positions within the phantom, with these locations validated by extracting intensity data using specialized software, as shown in Figure 5b,c. The intensity data were plotted against the distance within the field of view, revealing intensity variations that correlated with the tracking locations, as depicted in Figure 5d. The maximum values of the intensity data highlight the microrobot's center of mass or regions with the highest iron concentration. More motions of microrobot in porcine brain tissue are also investigated, the MPI‐overlayed optical images are shown in Figures S4 and S5 and Video S3 (Supporting Information). We then explored the potential of actuating and imaging the TriMag microrobot in biological fluids within an organ. Intravitreal delivery of therapeutic agents is critical for treating diseases such as diabetic retinopathy, glaucoma, and diabetic macular edema.^[^ ^88^ ^]^ To evaluate the feasibility of TriMag for ophthalmic applications, we investigated whether the microrobot could be actuated and guided through the vitreous body of the eye. Using a porcine eye model, the microrobot was injected into the vitreous humor (Figure 5e). It was then actuated under a rotating magnetic field enhanced by a gradient.^[^ ^24^, ^39^, ^41^ ^]^ Our results demonstrate that when the microrobot was introduced near the center adjacent to the ocular wall, it actively migrated toward the fundus within 30 min under magnetic actuation, supporting the potential for targeted delivery in potential clinical applications (Figure 5f; Video S4, Supporting Information). These findings highlight the microrobot's promise for minimally invasive interventions in ophthalmology. A blood hemolysis assay is performed to verify the safety of the microrobots, and hemolysis levels below 5% are observed (Figure S7, Supporting Information, Experimental Section), indicating the safety of the microrobots.

We further evaluated the capability of actuating and imaging the TriMag microrobot in live animal models. Figure 5g illustrates the schematic of an in vivo experiment designed to visualize and guide the microrobot's motion within the stomach of a mouse using magnetic particle imaging (MPI) with CT co‐registration. The microrobot was administered orally via gavage into the stomach. To improve the CT imaging contrast of the stomach, a contrast agent containing 10% barium sulfate (BaSO_4_) and 0.5% mannitol was used as the liquid for the oral administration of the microrobot. Once inside, an external rotating magnetic field was applied to control the microrobot's movement toward designated target regions, simulating a typical gastric navigation path. The controlled motion followed a “C”‐shaped trajectory across the curvature of the stomach, mimicking procedures relevant for clinical interventions and bioelectronic device delivery. MPI and CT co‐registered images captured the anatomical shape of the stomach and the real‐time position of the microrobot. A time‐series MPI study further demonstrated the dynamic movement of the microrobot: it traveled from the greater curvature, approached the pylorus, and eventually returned to the fundus region (Figure 5h; Video S4, Supporting Information). Due to the high viscosity of the environment, the microrobot's motion was quasi‐equilibrium, providing enough operation time to actuate and control the robot. At various stages, the microrobot body was observed with well‐defined edges, providing the imaging process is reliable. These imaging techniques facilitate non‐invasive monitoring of microrobots, offering significant potential for various biological and medical applications.

### 2.6 Magnetothermal Heating

**Figure 6:**
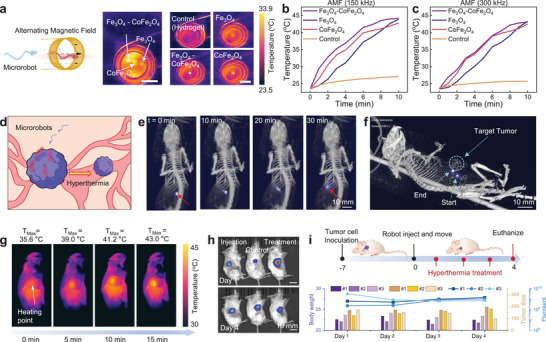
Magnetothermal heating using the TriMag microrobots. a) Schematic of the heating experiment using hydrogels with different magnetic nanoparticles. Scale bar = 15 mm. b,c) Temperature increase of hydrogels with different magnetic nanoparticles, measured by an optical thermometer. The remote heating frequencies are (b) 150 kHz and (c) 300 kHz magnetic fields. d) Schematic illustration of a heated microrobot designed for localized tumor ablation. e,f) The CT‐MPI tracked microrobots in the environment of the living mouse in (e) 2D mode and (f) 3D mode. The microrobot is injected into the ambient position of the tumor, then rotated and dragged by the magnetic field to the peripheral area of the tumor, and the moving distance is approximately in the range of 5–10 mm. The pictures in (e) were tracked every 10 min. Scale bar = 10 mm. g) Time‐series images captured by a thermal camera, showing the local temperature increase process every 5 min. Scale bar = 15 mm. h) Longitudinal IVIS fluorescence imaging on Day 0–4 shows microrobot accumulation and reduced tumor signal post‐treatment. Scale bar = 15 mm. i) The overall workflow of in vivo tumor hyperthermia treatment. Tumor cells were inoculated 7 days prior to microrobot injection. Then the TriMag microrobot was magnetically actuated from the injection site to the tumor location, followed by localized heating using an external alternating magnetic field. The quantitative evaluation of therapeutic outcomes includes the changes in body weight, tumor volume, and fluorescence intensity over the 4‐day treatment period.

One application of magnetic nanoparticle‐based structures is their ability to harness the magnetothermal effect of paramagnetic materials. This property allows the microrobot in this study to be remotely heated by a high‐frequency time‐varying magnetic field. To evaluate this effect, we first tested the magnetothermal properties of different materials in an in vitro environment. Hydrogel samples composed of three materials (Fe_3_O_4_, CoFe_2_O_4_, and a Fe_3_O_4_‐CoFe_2_O_4_ combination, same as Figure 4) were placed in the center of a custom‐built magnetothermal heat generator (Figure S6, Supporting Information), capable of producing an oscillating magnetic field at frequencies between 150 and 300 kHz. A fiber‐optic temperature sensor (TS2‐02, MICRONOR) was inserted into each hydrogel sample, and temperature measurements were recorded using an optical fiber thermometer (FT‐MNT, OPTOCON). Thermal images (FLIR one pro, Teledyne FLIR, Oregon, US) in **Figure** **6**a show the temperature difference of different samples after 90 s of heating with a frequency of 150 kHz and a magnetic field of 21.5 mT, indicating that the hydrogel with Fe_3_O_4_‐CoFe_2_O_4_ nanoparticles reached a higher temperature. Subsequently, the hydrogel samples were remotely heated using 150 and 300 kHz magnetic fields for 10 min, with the temperature measured by the thermometers illustrated in Figure 6b,c, respectively. The Fe_3_O_4_‐CoFe_2_O_4_ combination material exhibited the fastest temperature rise from baseline to the typical therapeutic threshold (41 °C) compared with Fe_3_O_4_ and CoFe_2_O_4_, showing its great potential in hyperthermia treatment applications.^[^ ^85^, ^89^, ^90^ ^]^ These results suggest that the Fe_3_O_4_‐CoFe_2_O_4_ combination material could significantly reduce operation time, making it advantageous for magnetic hyperthermia therapy applications.

We next investigated the potential of microrobotic hyperthermia therapy using a murine tumor model. Prior studies have demonstrated that actively propelled microrobots can serve as highly effective tools for physically targeting tumor tissues, providing a promising alternative to conventional chemotherapy, which often leads to significant systemic side effects.^[^ ^90^ ^]^ For tumors located in anatomically challenging or protected regions, such as those behind the blood–brain barrier, theshort‐range, targeted strategies offer distinct advantages. Compared to traditional local delivery methods such as intratumoral injection, which can be highly invasive and unsuitable for repeated use, untethered microrobots provide a minimally invasive solution. Their active navigation capability enables precise delivery to deep‐seated tumors while minimizing collateral tissue damage.

In this study, we explored a strategy distinct from passive diffusion‐based subcutaneous delivery. Following peritumoral injection, the magnetically actuated microrobots were shown to actively penetrate through dense subcutaneous tissue, increasing local agent concentration while minimizing uncontrolled diffusion and therapeutic loss.^[^ ^6^ ^]^ Furthermore, the magnetothermal effect generated by the microrobots can locally ablate adipose tissue and connective protein networks, thereby reducing mechanical resistance and accelerating microrobot propulsion in complex tissue environments.^[^ ^91^ ^]^ Based on these considerations, we designed an in vivo experiment to validate the feasibility of targeted microrobotic hyperthermia therapy. As illustrated in Figure 6d, microrobots were peritumorally injected and magnetically guided toward the extracellular tumor matrix using a rotating magnetic field operating at 2 Hz. Upon arrival, an alternating magnetic field (150 kHz) was applied to trigger localized heating via magnetothermal effects. The heat was confined to the targeted region, thereby reducing the risk of thermal injury to surrounding healthy tissues. According to prior studies, the effective concentration of hyperthermia agents typically ranges from several milligrams per gram of tumor tissue.^[^ ^92^, ^93^ ^]^ Based on this, we administered a 0.1 mL injection containing microrobots equivalent to 5 mg mL^−1^ of magnetic nanoparticles.

CT and MPI images in Figure 6e,f visualize the microrobot's motion in both 2D and 3D. In Figure 6e, the microrobot is shown actively migrating from the injection site to the tumor matrix within 30 min, demonstrating the feasibility of guided delivery in the peritumoral region. Once localized at the tumor site, hyperthermia therapy was initiated. Time‐series thermal images (Figure 6g) show a progressive increase in temperature during the 30‐min treatment session. By the 15‐min mark, the microrobot's local temperature increased from physiological baseline to 43 °C, a range effective for tumor ablation while remaining below the threshold for thermal injury to normal tissue. To ensure safety, the magnetic field was automatically switched off when the skin temperature approached 45 °C.

Therapeutic outcomes were evaluated across three treatment groups: no treatment (PBS injection), microrobot injection only, and full hyperthermia therapy. As shown in Figure 6h, the untreated and injection‐only groups exhibited no measurable tumor reduction, with fluorescence signals remaining constant or even increasing relative to baseline. In contrast, the hyperthermia‐treated group showed a dramatic decrease in tumor fluorescence intensity within 1–2 days post‐treatment, indicating rapid liquefaction and resorption of tumor tissue. Importantly, treated animals maintained normal locomotion with no signs of limb dysfunction, suggesting that the thermal treatment was well localized and caused minimal damage to surrounding tissues. The complete workflow of the in vivo microrobotic hyperthermia treatment is summarized in Figure 6i. Quantitative evaluation of therapeutic outcomes, including body weight, tumor volume, and fluorescence intensity, was conducted over a 4‐day period (Figure 6i; Figure S8, Supporting Information). The results demonstrate that magnetothermal hyperthermia significantly accelerated tumor size reduction, with animals maintaining stable body weight and exhibiting no observable off‐target toxicity. Together, these findings confirm both the efficacy and biocompatibility of the treatment protocol and illustrate the clinical potential of the TriMag microrobot system for targeted, minimally invasive cancer therapy.

## 3 Conclusion

In this study, we demonstrated a novel approach to fabricating magnetic microrobots by combining two‐photon lithography and in situ chemical reactions within a hydrogel matrix. This advanced fabrication method enables scalable and precise production of magnetic hydrogel microrobots, embedded with Fe_3_O_4_ and CoFe_2_O_4_ nanoparticles to achieve triple magnetic functionalities. These microrobots exhibit efficient magnetic actuation, precise magnetic particle imaging (MPI), and magnetothermal heating, showcasing their potential for minimally invasive interventions in biological tissues. The incorporation of superparamagnetic nanoparticles not only enhances magnetic functionality but also enables accurate detection and localization of the microrobot. Using a 3D electromagnetic system, precise actuation control was achieved. Additionally, the application of MPI provided high‐resolution imaging capabilities, allowing real‐time tracking of the microrobots’ positions, even in deep tissue environments, where conventional imaging techniques often face challenges. The ex vivo experiments further illustrated the capacity of MPI tracking of microrobots in the deep brain and the ability to achieve wireless magnetothermal heating, which could be used for various hyperthermia or biological stimulation applications. Overall, the successful demonstration of these multifunctional microrobots highlights their potential for precise biomedical applications, including targeted drug delivery, localized hyperthermia, and advanced imaging in complex tissue environments.

## 4 Experimental Section

### Materials

All the materials used in this research were purchased from Sigma‐Aldrich unless otherwise noted. The Polyethylene glycol diacrylate used in the experiments is PEGDA‐250 (Thermo Scientific, MA, US). Phenylbis(2,4,6‐trimethylbenzoyl) phosphineoxide (BAPO) and isopropylthioxanthone (ITX) were used as photoinitiator and photoabsorber, respectively. The BAPO and ITX were dissolved using Polyethylene glycol diacrylate in a weight ratio of PEGDA/BAPO/ITX = 70:0.8:0.5, then slowly mixed with PETA (weight ratio: 28.7) to make the photosensitive precursor without functional components (Fe_3_O_4_ and CoFe_2_O_4_).

The functional ion will be provided by iron(II) sulfate heptahydrate, iron(III) chloride, and cobalt(II) chloride hexahydrate. For the Fe_3_O_4_‐CoFe_2_O_4_ combination material, the three components were dissolved in DI water with a weight ratio of iron(II)/iron(III)/cobalt(II)/Water = 0.1/0.4/0.1/1, then added to 3x propylene glycol. After being well mixed by 2 h of ultrasonic and 12 h of blender, the functional component could be added to the photosensitive precursor to make 3D printing ink for TPP. To improve the generated particle performance, a trace amount of poly(vinyl alcohol) will be helpful.

In order to improve the fabrication and removal performance between the microrobot and glass substrate, a glucose sacrificial layer could be fabricated before 3D micro‐printing processes (10% glucose in DI water, 1500 rpm for 1 min). Once the printing was complete, the microrobot was removed from the printing bed and sonicated to remove any excess ink for 10 min in isopropanol alcohol and 2 min in water, then stored in functional solvent if not going to be used immediately.

### Magnetothermal Experiment Setup

The microrobot is remotely heated using a custom‐made magnetic hyperthermia system, which generates high‐frequency alternating magnetic fields to heat the CoFe_2_O_4_ and SPIONs components within the microrobot via the magnetothermal effect. The device operates within a frequency range of 100 kHz–0.5 MHz, with the frequency set at 250 kHz for this study. The system consists of a high‐power DC supply, water‐cooled solenoid, self‐excited oscillator, onboard amplifier, and pumping system. This magnetic hyperthermia setup can be seamlessly integrated with the Magnetic Particle Imaging (MPI) system by positioning the solenoid at the entrance of the MPI scanning chamber. Further technical details are provided in the Supporting Information.

### Distributed Optical Fiber Temperature Measuring System (DTS)

In this research, the DTS system was adopted as the temperature measurement system to eliminate the effect of any metallic material within the time‐varying magnetic field environment. The system functioned by converting thermal signals into optical signals through the TS2 fiber optic temperature probe (Model TS2‐02, Micronor Sensors, CA). These optical signals were then collected and processed by a single‐channel fiber optic signal conditioner PCB module (Model FOTEMP1‐OEM‐MNT, Micronor Sensors, CA).

### Mouse Oral Gavage Delivered Microrobot and Tumor Xenografts

c57 mice in clean grade, male, 6–8 weeks old, were provided by Michigan State University; all the experiments were performed in accordance with relevant guidelines and regulations. All mice were housed in a controlled environment, the room temperature is controlled at 23–25 °C, humidity is 50% ± 10%, provided a well‐ventilated environment and 12‐h light/dark cycles. Animals were acclimatized to the environment for 1 week with free access to a standard pellet diet before initiation of the experiments. For the microrobot in stomach experiment, the mice should be moved to an isolated cage with only tap water for ≈ 12 h prior to the enema operation. The microrobot is placed in 1.5 mL contrast agent solvent (10% BaSO_4_ and 0.5% Mannitol in 1x PBS), ejected into the stomach using a flexible esophageal feeding tube. The mice will be sacrificed when the experiment is finished.

The mice used in the tumor treatment experiment follow the same pre‐process as the in vivo experiment in stomach. Triple‐negative breast cancer cell lines (luc2‐expressing 4T1 cells) were collected, centrifuged, and resuspended in PBS. 4T1 cells (1 × 10^6^ cells per mouse) were inoculated into 3 female Balb/c mice (Charles River Breeding Laboratories). Orthotopic tumor‐bearing mice were considered ready for in vivo studies when the tumor volume reached >100 mm^3^. The tumor volume was calculated by the following equation: V = W^2^ × L/2, where W and L are the width and length, respectively, of the tumor measured by calipers. Mice were restored to their own cages after the operation (injection, thermal treatment, etc.). Survival and health status were monitored until the day of euthanasia. All the tumors will be kept separately for further research. Ethical approval was obtained from the Ethics Committee at Michigan State University (Approval No. PROTO202200377 and No. PROTO202100307). All animal care and experimental procedures were conducted in accordance with the guidelines set by Michigan State University.

### Microrobot Actuation/Observation in Porcine Eyes

Porcine eyes were obtained from Animal Technologies, Texas, and stored in ice for transportation. The samples for the microscope propulsion experiments were dissected from the porcine humor and used within 30 min after being defrosted. The sample was placed at the center of two orthogonal electromagnetic coils and observed under a microscope. To increase the propulsion force, a 2‐inch cube magnet could be placed under the bottom of the eyeball, with a distance between the magnet and the eyeball was ≈10 mm. The driving magnetic field had a strength of 130 mT, and the rotational frequency was swept from 2 to 10 Hz for propulsion by adjustment.

### In Vivo Imaging System (IVIS)

Bioluminescence imaging to track tumor response was conducted by intraperitoneally injecting D‐luciferin (150 mg kg^−1^) and imaging on an IVIS (PerkinElmer, USA. Subject height: 1.5 cm) every day (4 days in total). The luciferin was injected intraperitoneally (i.p.) or around the tumor 10 min before imaging.

### Hemolysis Assay Experiment

The fresh blood was collected from 3–4‐month mice. Centrifuged fresh blood with PBS at 1000 rpm for 5 min to remove plasma and buffy coat, then washed the red blood cell (RBC) pellet with PBS 3 times until the supernatant was clear, followed by resuspending the RBCs to a 2% (v/v) working suspension in PBS. Next, mix 100 µL of microrobot suspension (one robot for each) with 100 µL of 2% RBC suspension, incubate all samples at 37 °C for 1 h. For the supernatant collection process, centrifuged samples at 1000 rpm for 10 min, collected the supernatant into 96‐well plate, and then initiated the absorbance measurement at 540 nm by SpectraMax M3 plate reader (Molecular Devices, San Jose, CA). In this experiment, the positive control group was 100 µL RBC with 100 µL 0.1% Triton X‐100, the negative control group was 100 µL RBC with 100 µL PBS, experiment groups were microrobot with normal magnetization, microrobot without magnetization, and magnetic material including Fe_3_O_4_ and CoFe_2_O_4_. Statistical differences were determined by one‐way ANOVA, and results were expressed as mean ± SD. *p*‐Value < 0.05 was considered to indicate statistical significance. Statistical analyses were performed using GraphPad Prism 7 (GraphPad Inc.).

## Conflict of Interest

The authors declare no conflict of interest.

## Supporting information



Supporting Information

Supplemental Video 1

Supplemental Video 2

Supplemental Video 3

Supplemental Video 4

## Data Availability

The data that support the findings of this study are available from the corresponding author upon reasonable request.
