# Supplementary material for: TriMag Microrobots: 3D‐Printed Microrobots for Magnetic Actuation, Imaging, and Hyperthermia
Source: Adv Mater. 2025 Aug 11;37(45):e19708. doi: 10.1002/adma.202419708 (PMC12617050; doi:10.1002/adma.202419708)
Supplement: Supplementary file 1 — Supporting Information [file ADMA-37-e19708-s001.pdf]

ADVANCED MATERIALS

Supporting Information

for *Adv. Mater.*, DOI 10.1002/adma.202419708

TriMag Microrobots: 3D-Printed Microrobots for Magnetic Actuation, Imaging, and Hyperthermia

*Liuxi Xing, Yulu Cai, Yapei Zhang, Kevin Mozel, Zhengxu Tang, Tengteng Tang, Vittorio Mottini, Saumya Nigam, Bryan R. Smith, Ian Y Lee, Tavarekere N. Nagaraja, Ping Wang, Xiangjia Li, Tong Gao and Jinxing Li**

Supplementary Information

TriMag Microrobots: 3D-Printed Microrobots for Magnetic Actuation, Imaging, and Hyperthermia

*Liuxi Xing[+], Yulu Cai[+], Yapei Zhang, Kevin Mozel, Zhengxu Tang, Tengteng Tang, Vittorio Mottini, Saumya Nigam, Bryan R. Smith, Ian Y Lee, Tavarekere N. Nagaraja, Ping Wang, Xiangjia Li, Tong Gao, Jinxing Li**

L. Xing, Y. Cai, K. Mozel, Z. Tang, V. Mottini, Y. Zhang, B. R. Smith, J. Li
Department of Biomedical Engineering and Institute for Quantitative Health Science and Engineering, Michigan State University, East Lansing, MI, United States
E-mail: jl@msu.edu

T. Tang, X. Li
Department of Aerospace and Mechanical Engineering, Arizona State University, Tempe, AZ, United States

T. Gao
Department of Mechanical Engineering, Michigan State University, East Lansing, MI, United States

I. Y. Lee, T. N Nagaraja, Henry Ford Hospital, Detroit, MI, United States

S. Nigam, P. Wang
Precision Health Program, Michigan State University, East Lansing, MI, United States
Department of Radiology, College of Human Medicine, Michigan State University, East Lansing, MI, United States

[+]These authors contributed equally.

This PDF file includes:

Fig. S1. **Hydrogel stability testing experiment.**
Fig. S2. **SEM and EDS mapping images**.
Fig. S3. **Magnetic actuation system setup**
Fig. S4. **Optical enhanced MPI imaging of microrobot in vasculature-mimicking blood phantom**.

Fig. S5. **The MPI tracked microrobot in S-shape tube environment**.
Fig. S6. **Magnetic oscillation system for magnetic hyperthermia experiment.**
Fig. S7. **In vivo fluorescence imaging of tumor site from Day 1 to Day 4 using IVIS.**
Content: Microrobot system design and actuation

Other Supplementary Materials for this manuscript includes:

Movie S1. **Demonstration of in situ chemical reaction**.
Movie S2. **Microrobot actuation experiment**.
Movie S3. **Snapshot of in vivo microrobot motion using CT-MPI**
Movie S4. **Movie showing a microrobot moving in a pig eye and a mouse stomach**

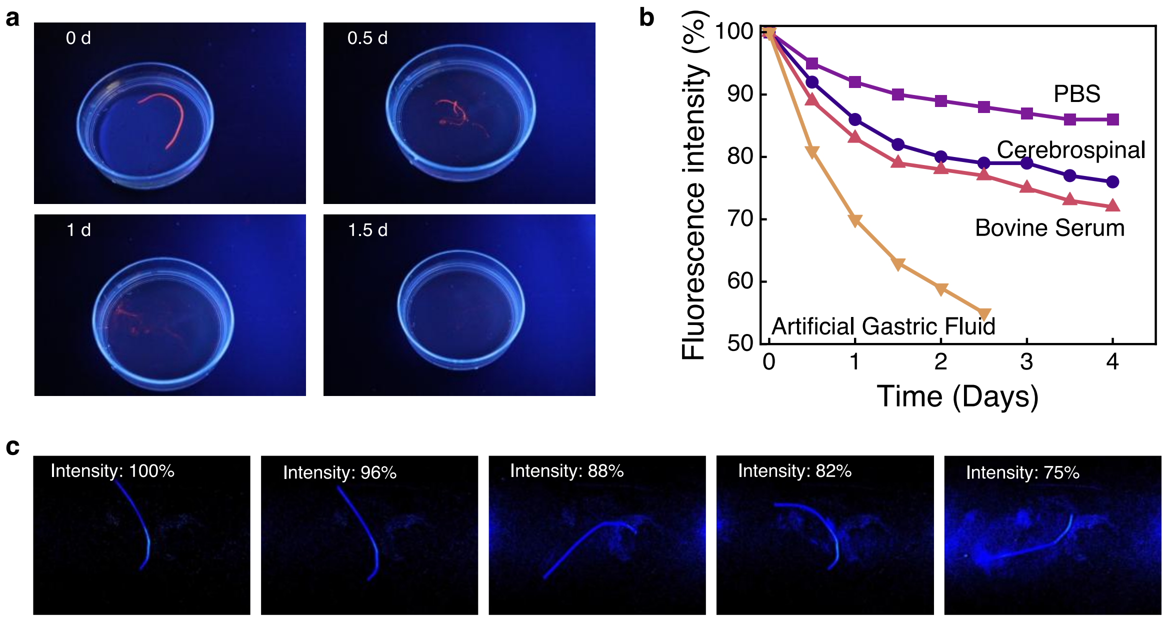

Fig. S1. Hydrogel degradability evaluation. To assess hydrogel degradation, 1% w/w fluorescent particles were mixed into the hydrogel precursor solution during fabrication. As the hydrogel degrades in liquid environments, embedded fluorescent particles are released, providing a visual and quantitative indication of degradation progress. (a) Fluorescently labeled hydrogel structures were immersed in a custom-made degradation solution (NaOH in PBS). Images were captured every 12 hours to monitor changes in hydrogel integrity. (b) Quantitative analysis of fluorescence intensity over a 4-day period was conducted in various biologically relevant media, including deionized water, 1% PBS, artificial cerebrospinal fluid (aCSF), bovine serum, and artificial gastric fluid. The hydrogel demonstrated the highest stability in 1% PBS, with progressively faster degradation observed in aCSF, bovine serum, and artificial gastric fluid. Artificial gastric fluid was composed of 0.2% (w/v) sodium chloride in 0.7% (v/v) hydrochloric acid. aCSF and bovine serum were obtained from Bio-Techne (MN, USA). (c) Schematic of the fluorescence-based quantification method. Fluorescently labeled hydrogels were placed in Petri dishes, and fluorescence intensity was measured at designated timepoints. During each measurement, the hydrogel was manually transferred to a clean imaging chamber to avoid signal contamination, then returned to the original dish. If the hydrogel became too small or fragmented, the measurement process was discontinued.

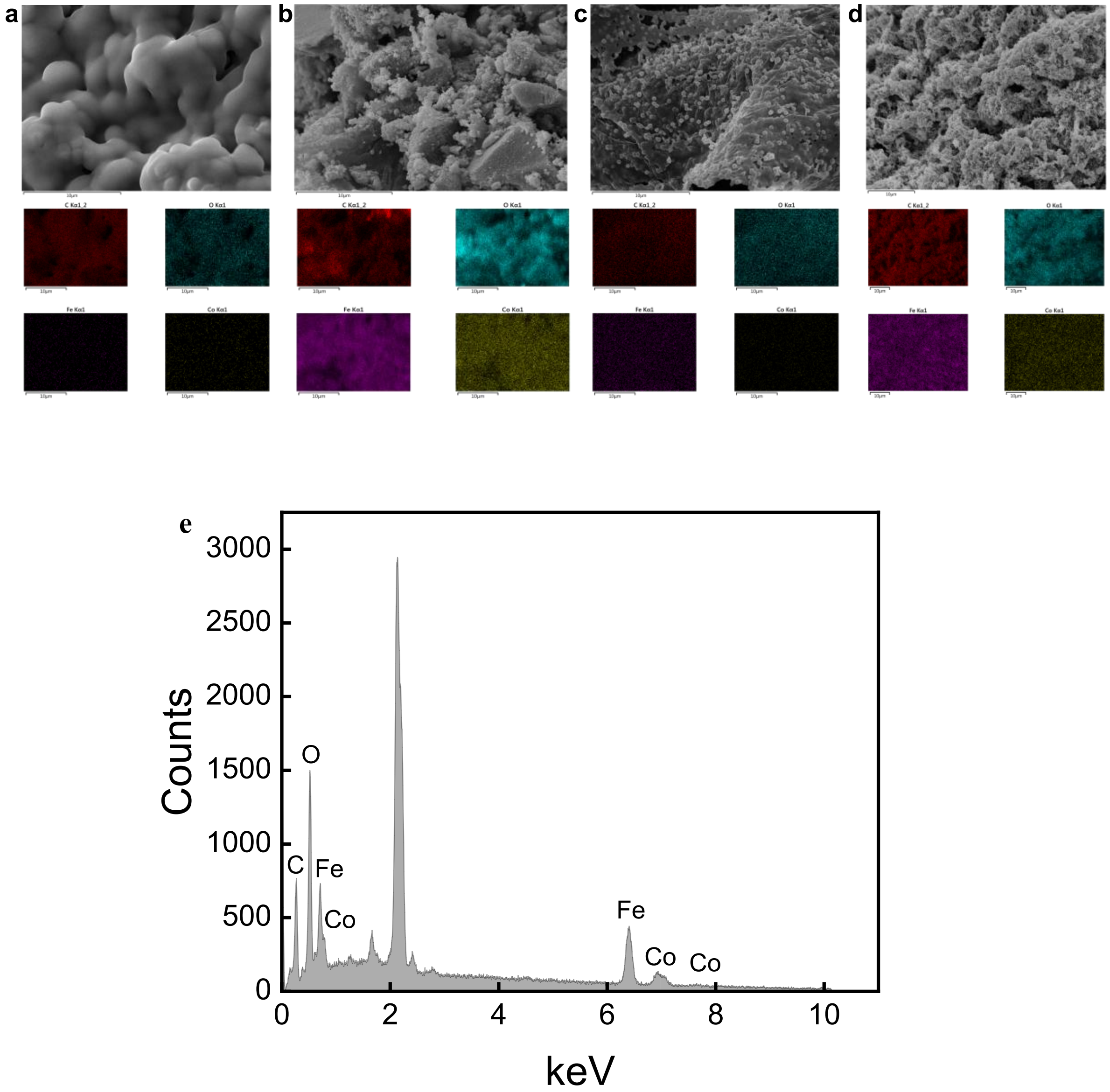

Fig. S2. SEM and EDS mapping images illustrate four distinct samples with varying compositions and structures. (a) The SEM image of pure gel. Elemental mapping reveals a predominant presence of C (Carbon) and O (Oxygen), suggesting that this region consists of a pure gel material without any added magnetic particles. **(b)** The SEM image of chemical particles only. The elemental maps show significant signals for Fe and Co, along with Oxygen, indicating the presence of Fe_3O_4 and $CoFe_2O_4$ magnetic particles. (**c**) The SEM image of magnetized gel. Elemental mapping confirms the presence of Fe and Co detected. This suggests Fe_3O_4 and $CoFe_2O_4$ magnetic particles embeded in the gel matrix. (**d**) The SEM image of crushed magnetized gel shows inside topography, along with extensive particle aggregation. Elemental mapping further confrmed mixed distribution of Fe_3O_4 and $CoFe_2O_4$ magnetic particles throughout the gel. (**e**) The energy-dispersive X-ray (EDX) spectrum confirms the presence of cobalt (Co), iron (Fe), oxygen (O), and carbon (C) elements in the sample.

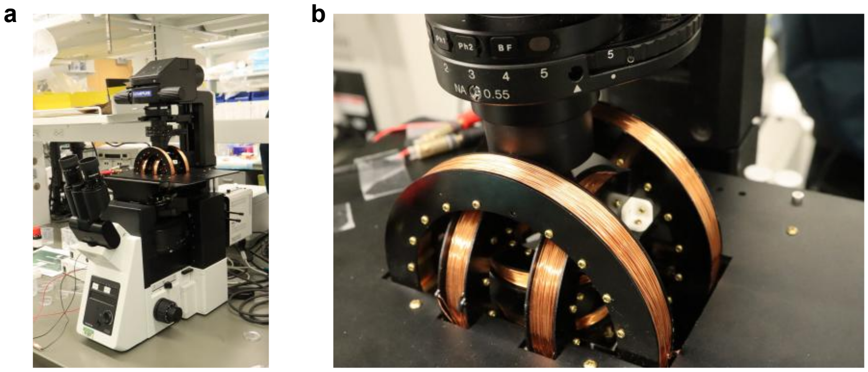

Fig. S3. Magnetic actuation system setup for the microrobot trajectory control experiment. **(a)** Three axis magnetic coils assembled in the microscopy platform. (**b**) The close-up view of the three-axis Helmholtz coils.

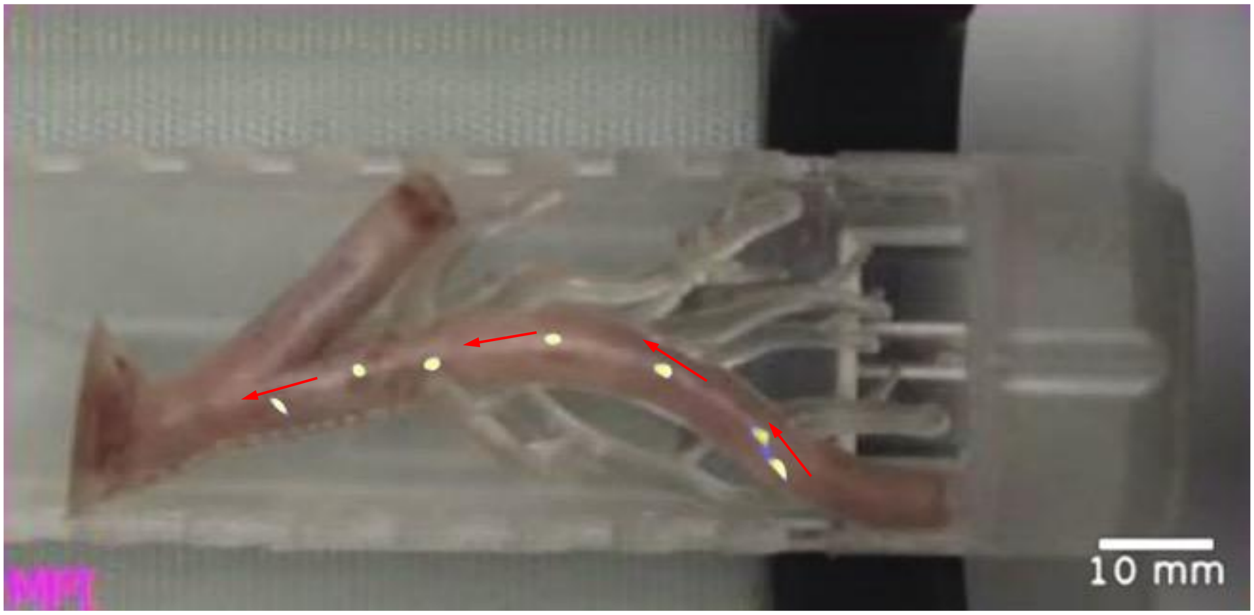

Fig. S4. The optical enhanced MPI imaging of microrobot in vasculature-mimicking blood phantom.

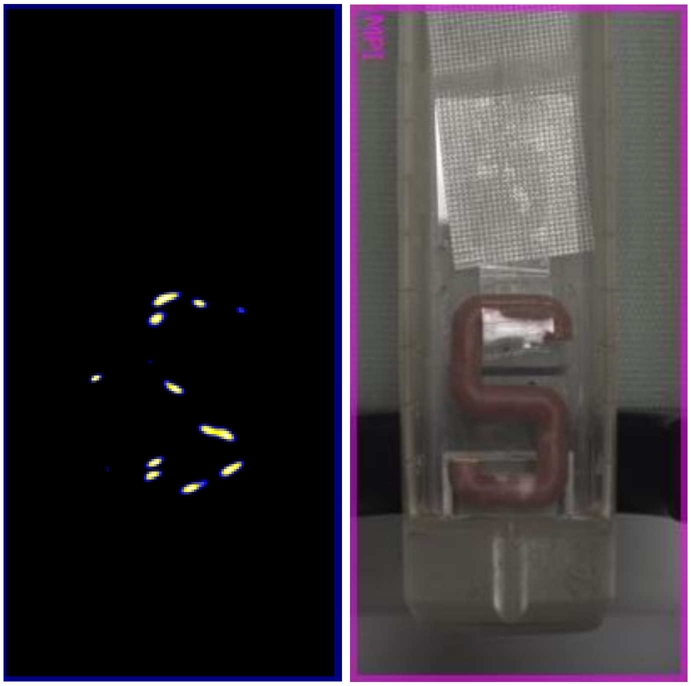

Fig. S5. The MPI tracked microrobot in S-shape tube environment.

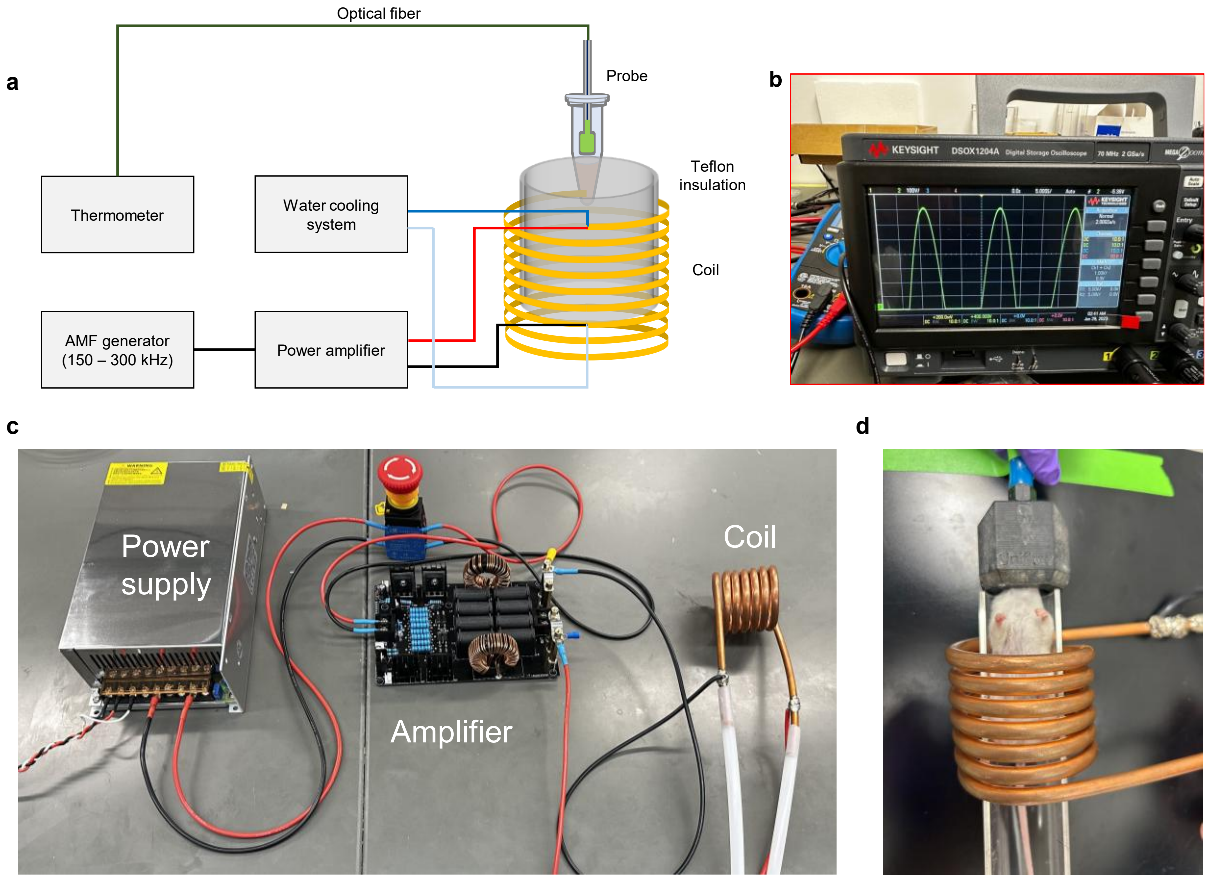

Fig. S6. Magnetic oscillation system for magnetic hyperthermia experiment. (**a**)The diagram of the magnetic oscillation system, including the AMF signal generation and amplification, water cooling system, and the optic thermometer to avoid the affection from metal. (**b**) The AMF wave measurement shows the actuation signal of the coil system. (**c**) The picture of AMF system. Water pump is not in this picture. (**d**) The hyperthermia tumor treatment. During the process, the mice is under anesthesia.

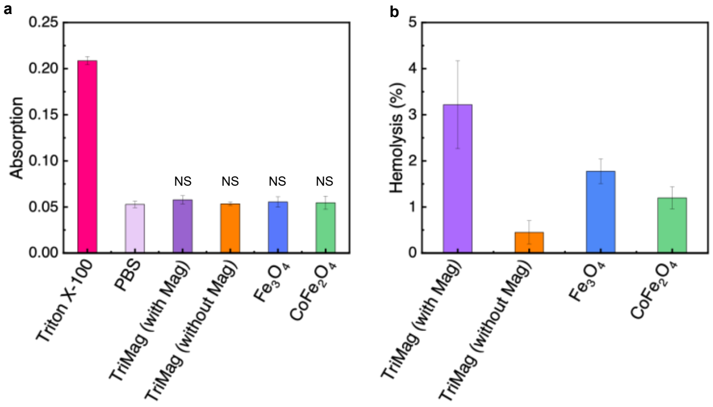

Fig. S7. Hemolysis assay testing for long-term safety verification. The microrobot and its components are added to the fresh blood from mice to test the safety of the microrobot. The result of (a) absorption and (b) hemolysis assay indicate that the blood compatibility of the microrobots is acceptable (All the quantify hemolysis < 5%) The positive control group is Triton X-100, negative control group is pure PBS, experiment groups are microrobot with magnetization, microrobot without magnetization, magnetic material including Fe_3O_4 and $CoFe_2O_4$. Each group was compared with PBS group, NS: No Significant difference.

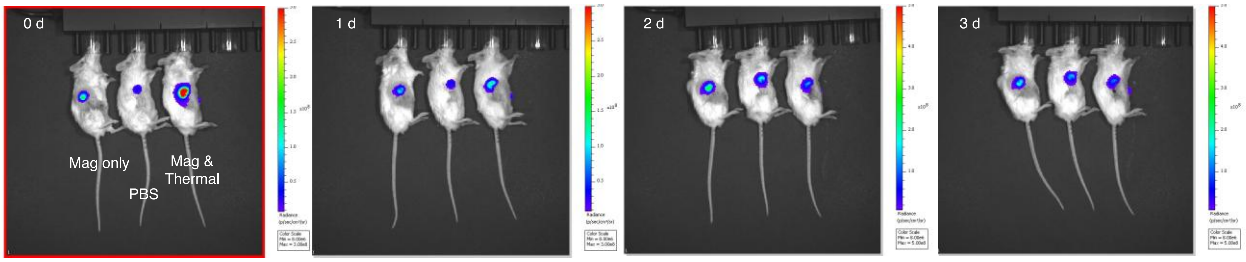

Fig. S8. In vivo fluorescence imaging of tumor site from Day 1 to Day 4 using IVIS. Fluorescence signals were collected daily following injection of the fluorescent sustance (Luciferin, 150 mg/kg) into tumor-bearing mice. Imaging was performed 10 minutes post-injection using the IVIS Spectrum system. Data were normalized, min (8×10^6), max (3×10^8)

Content: Microrobot system design and actuation

1. Magnetic actuation system

The study utilized helical microrobots that were actuated using either a 3D electromagnetic system known as a Helmholtz or neodymium permanent magnets. The microrobots contain superparamagnetic material after chemical reaction, which will be affected by a magnetic field, resulting in torque caused by the interaction between the controlled magnetic field and the average magnetization, as illustrated in equation 1.

$$T = \mu_0 \vartheta M \times H \qquad (1)$$

In this formula, T represents the generated torque that allows for movement in a particular direction. The constant μ_0 signifies the permeability of free space, with a value of $4\pi \times 10^{-7}$ Tm/A. The variable ϑ denotes the helix's volume in cubic meters, while M stands for the system's average magnetization, and H refers to the controlled magnetic field. This formula can also be expressed in terms of the applied magnetic field by replacing H with B, which symbolizes the applied magnetics flux density.[3,4] This conversion can be achieved using the relationship $B = \mu_0 H$. The assumptions made in this context include that the H value is applied to the helix's center of mass and that the applied field remains virtually uniform, unaffected by any spatial variations.[3,5] When employing a permanent magnet, it is reasonable to consider the average magnetization as a constant.[3] Additionally, the equation can be simplified by substituting M with the magnetic dipole moment, which is equal to the product of ϑ and M.[5]

Our findings revealed a maximum frequency at which the helix's rotation began to decelerate. This occurrence is due to insufficient torque to maintain the helix's synchronous rotation with the rotational magnetic field. Furthermore, we monitored the mean velocity along each trajectory that the microrobot followed. The experiments were carried out in both viscous and non-viscous environments, yielding distinct outcomes since viscosity influences an object's velocity.

2. Helical robot design

Helix microrobots used in this research are small, cylindrical robots that are capable of moving in a rotational manner. The robots in this research works in two different motions, named rolling motion and propulsion motion [6,7]. For the rolling motion, it is defined that the robots rotate along the axis which is vertical to its body axis, just like an athlete practicing the somersault motion. For the propulsion motion, the movement technique defined and employed in this research involved

progressing along the helix's axis. This motion was made possible by using a rotating field within an electromagnetic coil. By alternatively adjust the current in the magnetic coils, the orientation of the overall field will be rotated with designed logic.

The rolling motion is much more straightforward to be analysis, where the motion speed is equal to the rotating frequency multiply by the body length. The propulsion motion of helical robot was modeled based on two main parameters: helix angle and the ratio between length and diameter of the device. The helix angle was optimized to be as close to 45 degrees as possible, as this configuration has been demonstrated to exhibit the lowest misalignment angle[5]. The helix angle was calculated using Equation 1, which takes into account the helix radius and pitch length. The Helix Angle equation is used to determine the misalignment angle of the helix, which can cause it to rotate on the wrong axis [3]. This occurs when the axis of rotation is not aligned with the direction of propulsion, leading to inefficient movement.

$$Helix\ Angle = arctan\left(\frac{2\pi r_m}{L}\right) \quad (2)$$

This equation calculates the helix angle that is used to determine the misalignment angle of the helix. rm is the radius of the helix that is multiplied by two pie to determine the circumference. L is the length of the pitch which is the length between one full rotation of the helix.

3. Summary

The helical (or corkscrew) microrobot structure has been a foundational design in the field of micro/nanoscale propulsion since it was first proposed over a decade ago. Inspired by the locomotion mechanism of flagellated bacteria such as E. coli, this geometry was initially introduced in pioneering works, where microscale helices fabricated via direct laser writing or rolled-up nanotechnology demonstrated effective propulsion under rotating magnetic fields. Since then, the helical body has been widely adopted as a versatile and reliable platform for magnetically actuated microrobots. Specifically, the adopted geometry offers three key advantages in this work:

- *Non-collinear Net Magnetic Moment Generation*: As an asymmetric structure, the geomatic of the helical structure will result net magnetic moment is intentionally misaligned with the body’s major axis. This design allows the swimmer to undergo rolling or corkscrew-like propulsion under rotating magnetic fields

- *Efficient Propulsion and Steering in Low Reynolds Media*: The helical body shape provides inherent mechanical asymmetry, which translates rotational input into forward motion with minimal motion complicity. This allows for controllable and energy-efficient propulsion, even within complex tissue environments or high-viscosity biofluids.
- *Optical Asymmetry for Pose Tracking*: The geometric asymmetry also permits the inclusion of localized optical or structural markers, enabling real-time position and orientation detection using MPI or hybrid MPI-CT imaging modalities. This feature is essential for feedback-based closed-loop microrobot control.

References

[1] T. Wei, J. Liu, D. Li, S. Chen, Y. Zhang, J. Li, L. Fan, Z. Guan, C.-M. Lo, L. Wang, K. Man, D. Sun, *Small* **2020**, *16*, e1906908.

[2] Z. S. Stillman, B. M. Jarai, N. Raman, P. Patel, C. A. Fromen, *Polym. Chem.* **2020**, *11*, 568.

[3] J. J. Abbott, K. E. Peyer, M. C. Lagomarsino, L. Zhang, L. Dong, I. K. Kaliakatsos, B. J. Nelson, *Int. J. Rob. Res.* **2009**, *28*, 1434.

[4] Y. Dong, L. Wang, V. Iacovacci, X. Wang, L. Zhang, B. J. Nelson, *Matter* **2022**, *5*, 77.

[5] S. Tottori, L. Zhang, F. Qiu, K. K. Krawczyk, A. Franco-Obregón, B. J. Nelson, *Adv. Mater.* **2012**, *24*, 811.

[6] L. Xing, D. Li, H. Cao, L. Fan, L. Zheng, L. Zhang, D. Sun, *Adv. Intell. Syst.* **2022**, 2100214.

[7] H. Xie, M. Sun, X. Fan, Z. Lin, W. Chen, L. Wang, L. Dong, Q. He, *Sci. Robot.* **2019**, *4*, eaav8006.
